# Advantages and Disadvantages of Medical Abortion, According to Brazilian Residents in Obstetrics and Gynaecology

**DOI:** 10.1055/s-0040-1718952

**Published:** 2020-12-21

**Authors:** Silvana Ferreira Bento, Karla Simônia de Pádua, Rodolfo de Carvalho Pacagnella, Karayna Gil Fernandes, Maria José Duarte Osis, Graciana Alves Duarte, Anibal Faúndes

**Affiliations:** 1 Universidade Estadual de Campinas, Campinas, SP, Brazil; 2 Centro de Pesquisas em Saúde Reprodutiva de Campinas, Campinas, SP, Brazil; 3 Faculdade de Medicina de Jundiaí, Jundiaí, SP, Brazil

**Keywords:** abortion, legal abortion, hospital medical staff, brazil, aborto, aborto legal, corpo clínico hospitalar, brasil

## Abstract

**Objective** To find out which was the opinion of residents in obstetrics and gynecology about the advantages and disadvantages of medical abortion as compared with surgical procedures.

**Method** Cross-sectional multicenter study among residents in obstetrics and gynecology from 21 maternity hospitals located in 4 different geographical regions of Brazil, using a self-responded questionnaire with 31 questions related to their opinion and experience on providing abortion services.

**Results** Most residents agreed that “being less invasive” (94.7%), “does not require anesthesia” (89.7%), “can be accompanied during the process” (89.1%), “prevents physical trauma” (84.4%) were the main advantages of medical abortion.

**Conclusion** Residents perceived both clinical and personal issues as advantages of medical abortion.

## Resumo

**Objetivo** Descobrir qual foi a opinião dos residentes em ginecologia e obstetrícia sobre as vantagens e desvantagens do aborto medicamentoso em relação aos procedimentos cirúrgicos.

**Métodos** Estudo multicêntrico transversal entre residentes de ginecologia e obstetrícia de 21 maternidades localizadas em 4 diferentes regiões geográficas do Brasil, utilizando um questionário autorrespondido com 31 questões relacionadas à sua opinião e experiência na prestação de serviços de aborto.

**Resultados** A maioria dos residentes concordou que "ser menos invasivo" (94,7%), "não necessitar de anestesia" (89,7%), "poder ser acompanhado durante o processo" (89,1%), "prevenir trauma físico" (84,4%) foram as principais vantagens do aborto medicamentoso.

**Conclusão** Os residentes perceberam tanto questões clínicas como pessoais como sendo vantagens do aborto medicamentoso.

## Introduction

Brazil has one of the most punitive abortion laws, similar as most of the countries in the Latin American region. 1 In spite of such legal restriction, it has one of the highest estimated incidence of abortions in the world 2 3 with ∼ 500,000 illegal abortions per year among women aged between 18 and 39 years old. 4 It is estimated that > 200 women die every year in Brazil as a consequence of unsafe abortions. 5 6

That situation has changed more recently, however, without any intervention of health authorities. Abortion related maternal mortality has been reduced in Brazil as in another Latin American countries, simply by the increasing utilization of misoprostol for pregnancy termination, by women themselves, instead of the more dangerous methods used earlier. 7 8

In parallel to those changes in the practice of illegal abortions, an effort to improve access to legal abortion was being carried out. Although the Brazilian penal code permits abortion to protect women's lives and in case the pregnancy is the result of rape, the practice of legal abortion in public hospitals was almost inexistent until very recently. It was almost 25 years ago that an initiative to facilitate access to abortion after rape was taken, calling to what was called Interprofessional Forum Sexual Violence and Abortion emitted by law, with participation of leading obstetricians and gynecologists from all over the country, as well as Ministry of Health authorities. 9

That initiative succeeded in stimulating the Ministry of Health to produce the norms for the care of women and adolescents who suffer sexual violence, including pregnancy termination when required. The first version was released in 1998, but it has been updated periodically, at least until 2010 _._ 10 The Interprofessional Forum on Sexual Violence and Abortion permitted by the law was repeated for several years, stimulating the increase in the number of teaching hospitals that provide legal abortion services around the country. 11

In spite of the norms and holding of repeated forums, the knowledge about the abortion laws and the requirements to provide safe, legal abortion services still remain poor. 12 It appears that the subject of abortion does not receive the same attention as other subjects, providing more technological information, which can serve for the improvements of the immediate care being provided to the patients, and consequently, the details of the law and on how to apply it remain practically ignored by a majority of Brazilian gynecologists.

In order to change that situation, it is fundamental to improve the training of the future specialists who are currently in their residency years. A key element that should be part of their training is about the procedures currently recommended for legal termination of pregnancy.

By now, there is general agreement that the emergence of drugs capable of inducing termination of pregnancy without any surgical intervention has led to improved access to safe legal abortion 13 or even to a less unsafe abortion when used by the women themselves. 14 15 16

The recommended medical method for pregnancy termination is the combination of an antiprogesterone originally called RU 486 and currently known as mifepristone, with a prostaglandin administered 24 to 48 hours later. 17 18

Recent very large studies strongly suggest that both surgical and medical methods are almost equally effective and safe. 19 The only important disadvantage of medical abortion is that it is not possible to administer a long acting contraceptive, such as an intrauterine device (IUD), immediately after the uterine evacuation, 20 as it occurs at home and the exact moment is unpredictable.

While misoprostol is available practically everywhere, in many countries mifepristone is not yet registered and not available, as it is the case of Brazil. Thus, the only form of medical abortion available in Brazil is misoprostol, which is registered only to be used by the vaginal route, and as such is recommended by the Ministry of Health itself for legal termination of pregnancy after rape. 21

Accordingly, it is supposed that Brazilian departments of obstetrics and gynecology in medical schools should be using misoprostol with that purpose in their teaching hospitals and should be teaching the subject in undergraduate and postgraduate training, more so to their residents in obstetrics and gynecology.

It was surprising, however, that in the same study on which the present article is based, we found that 20% said that they had not received any information on the use of misoprostol during residency, and almost 30% of those who received information judged that the information received on misoprostol was not sufficient for their needs _._ 22

This omission in the training of residents is an evident weakness in the teaching of the medical schools, but also suggest a lack of demand from the part of the residents.

In order to understand the reason for such lack of demand, we decided to evaluate which was the knowledge the residents had about the advantages and disadvantages of medical abortion with misoprostol, as compared with the use of surgical procedures.

## Methods

This was a cross-sectional multicenter study in which residents from 21 maternity hospitals with residency programs in obstetrics and gynecology, located in 4 different geographical regions of Brazil, responded a questionnaire with 31 questions related to their opinion and experience on providing abortion services.

The hospitals selected are part of the Brazilian Network for Studies in Reproductive and Perinatal Health. All these hospitals are obliged to provide safe abortion services to women who meet the conditions determined under the current legislation, and the residents are expected to participate in providing these services. Residents are able to participate in the care of women requesting legal abortion, including their admission, physical examination, etc., but they may not necessarily take part in performing the abortion. The involvement of the residents in providing safe abortion services was not necessarily the same in the different hospitals participating in the present study. A local supervisor was appointed at each of the participating hospitals. Each resident received a 31-item questionnaire to complete, although they were free to respond or not the full questionnaire or selected questions without being identified, as the completed (or not) questionnaire should be deposited in sealed boxes, which were opened only at the site and time of the analysis and there was no way to identify from which institution the box came or who had filled in each questionnaire. 22

The purpose of this procedure was to ensure the privacy of the participants by reassuring them that the supervisor would not know whether they had decided to participate or not in the study. The data collection process continued until all the residents had had the opportunity to participate in the study.

The questionnaire included items regarding sociodemographic characteristics of the participants, their knowledge regarding medical abortion, their personal and professional experiences related to abortion, their opinion on the advantages and disadvantages of medical abortion in comparison with surgical abortion, among several other items which are not part of this analysis. In the data analysis, the association between the personal experience of the residents in providing legal abortion services and their opinion related to advantages or disadvantages of medical abortion was evaluated using the chi-squared test.

To do so, the residents were divided into two groups according to their experience of being involved in the care of women requesting legal termination of pregnancy: those who had such experience and those who did not have been involved in providing legal abortion services. We performed the entire analysis using the R software package (R Foundation, Vienna, Austria). 23

The Institutional Review Board of the School of Medical Sciences of the Universidade Estadual de Campinas (UNICAMP, in the Portuguese acronym) approved the study protocol (CAAE: 21177013.3.0000.5404), as did the institutional review boards of each of the participating hospitals. The need for signed informed consent was waived considering the nature of the study and to ensure complete confidentiality.

## Results

### Characteristics of the Sample

At the time of the interview, there were 530 residents in obstetrics and gynecology in the 21 institutions included in the study, but only 83% were invited to participate in the survey and 77% returned the questionnaires (404 completed and 3 blanks). Seventy-one percent of the residents had been involved in providing legal abortion care to women. Most of them (327 or 81.1%) were female and 18.9% (76) were male. The majority did not have a stable partner at the moment of the interview (282; 70%), was < 27 years old (227; 56.3%) and referred that religion was very important (121; 36.8%) or important (176; 53.7%) in their lives ( Table 1 ).

**Table 1:** Sociodemographic characteristics of the respondents (n = 404)

Characteristics	n	%
Age (years old)	403	
≤ 27	227	56.3
≥ 28	176	43.7
Gender	403	
Female	327	81.1
Male	76	18.9
Marital Status	403	
Without a stable partner	282	70.0
With a stable partner	121	30.0
Year of residency	402	
1 ^st^ or 2 ^nd^	274	68.2
3 ^rd^ –5 ^th^	128	31.8
Region of birth		
Southeast	207	51.2
Northeast	117	29.0
North	35	8.7
South	28	6.9
Midwest	10	2.5
Another country	7	1.7
Declared religion *	403	
Catholic	235	58.3
Other	92	22.7
None	76	19.0
Importance of religion in their life	402	
Very important	121	36.8
Important	176	53.7
Of little or no importance	31	9.5

### Advantages and Disadvantages of Medical Abortion

There were five conditions which were considered advantages by between 84 and 95% of the respondents: “being less invasive” (94.7%), “does not require anesthesia” (89.7%), “can be accompanied during the process” (89.1%), “prevents physical trauma” (85.6%) and “is less expensive”(84.4%). In the other extreme, there were 4 conditions that were considered disadvantages by between 70 and 76% of the respondents: ”it has a higher failure rate” (76.2%), “it has more side-effects” (75.3%) and “bleeding may be greater” and “pain may be greater” (70.7% and 70.6%) respectively ( Table 2 ).

**Table 2:** General opinion of residents in obstetrics and gynecology on possible advantages and disadvantages of medical abortion compared with surgical abortion

Possible advantages/ disadvantages of medical abortion	Advantage %	Disadvantage %	Don't know %
Does not requires anesthesia (n= 398)	**89.7**	3.3	7.0
Women can see what is happening	51.3	29.7	19.0
More “natural”, like a miscarriage	79.4	6.6	14.1
Prevents physical trauma	**85.6**	4.0	10.3
Feeling in control of the process	60.0	10.3	29.8
It is less invasive	**94.7**	1.5	3.5
More private, can be done at home	40.8	34.7	24.6
Can be accompanied	**89.1**	3.0	7.8
Less expensive	84.4	4.5	11.1
Pain may be greater	13.8	**70.6**	15.6
Bleeding may be greater	10.4	**70.7**	18.8
Requires more visits	32.6	47.0	20.4
Higher failure rate	6.6	**76.2**	17.1
Women may have chills	6.8	68.2	24.9
It has more side effects	5.0	**75.3**	19.6

The only two advantages with a significantly higher percentage of agreement among the residents who had participated in legal abortion care were “feeling in control” and “being less invasive”. “Feeling in control” was considered an advantage by almost two thirds of the residents with experience in caring for women having legal abortions and by just less than half of the residents without such experience. The difference in the case of “being less invasive” was much smaller, although statistically significant. The difference in the proportion who agreed that “can be accompanied during the process” is an advantage was close to significance (0.058), with eight percentual points difference between those with and those without the experience of caring for women having a legal abortion ( Table 3 ).

**Table 3:** Advantages and disadvantages of medical abortion as compared with surgical abortion according to the resident's experience of being involved in providing legal abortion services

Possible advantages and disadvantages of medical abortion	Evaluation of residents	Involved in providing legal abortion services		
		Yes %	Not %	*p-value*
Did not require anesthesia (n= 398)	Advantage Disadvantage Don't know	91.3 0.9 7.8	89.1 4.2 6.7	0.219
Women can see what is happening	Advantage Disadvantage Don't know	52.8 28.5 18.7	47.4 32.8 19.8	0.599
More “natural”, like a miscarriage	Advantage Disadvantage Don't know	80.6 6.3 13.1	76.3 7.0 16.9	0.612
Prevent physical trauma	Advantage Disadvantage Don't know	97.2 3.6 9.2	81.7 5.2 13.1	0.366
Feeling in control of the process	Advantage Disadvantage Don't know	64.1 7.8 28.1	49.6 16.5 33.9	**0.008**
It is less invasive	Advantage Disadvantage Don't know	96.5 1.4 2.1	91.2 1.8 7.0	**0.043**
More private, can be done at home	Advantage Disadvantage Don't know	40.6 34.1 25.3	41.2 36.0 22.8	0.868
Can be accompanied during the process	Advantage Disadvantage Don't know	91.5 2.5 6.0	83.3 4.4 12.3	0.058
Less expensive	Advantage Disadvantage Don't know	85.4 4.6 10.0	81.7 4.4 13.9	0.525
Pain may be greater	Advantage Disadvantage Don't know	14.3 72.1 13.6	12.4 67.3 20.3	0.243
Bleeding may be greater	Advantage Disadvantage Don't know	11.1 71.3 17.6	8.8 69.3 21.9	0.524
Requires more visits	Advantage Disadvantage Don't know	31.0 48.0 21.0	36.5 44.4 19.1	0.563
Higher failure rate	Advantage Disadvantage Don't know	6.8 77.9 15.3	6.1 72.2 21.7	0.304
Women may have chills	Advantage Disadvantage Don't know	7.1 69.9 23.0	6.1 64.3 29.6	0.302
It has more side effects	Advantage Disadvantage Don't know	5.3 74.9 19.8	4.4 76.3 19.3	0.920

## Discussion

It was to be expected that the advantages on which a larger percentage of residents agreed were related to their clinical practice, such as “being less invasive”, “does not require anesthesia” and “prevents physical trauma”. These results agreed with an extensive review on women's perception of medical abortion in many different countries, 24 indicating that residents had a good evaluation of how women felt during this process.

It was interesting that a large percentage of residents also gave relevance to advantages for the women, such as “can be accompanied during the process” and “is more natural”, which were also perceived as advantage by women, as indicated by the review mentioned above. 24

Similarly, the disadvantages with higher percentage of agreement were also of very practical clinical relevance such as “it has higher failure rate” and “it has more side-effects”. The relevance given to the risk of greater incidence of bleeding and pain reflects that they are the most common complaint of women treated with medical abortion. 25

The only two advantages with higher percentage of agreement among the residents who had participated in legal abortion care reflects their better understanding of the feelings of the women who go through the process of having a pregnancy termination by the use of medications: “feeling in control”, and “being less invasive” are clearly advantages for the women involved and not for the providers. 24 These results seems to be a reflection of the closer proximity to the women under the care of the residents involved in providing such care. This is another example of how the clinical practice is important in creating a better client-provider interaction, which is a key element of good health care.

Another important discussion that can be raised by the results is regarding the use on an outpatient clinic basis. Although women with pregnancies up to 12 weeks of gestation can receive outpatient care for medical abortion, a scheme proven to be feasible and safe through outpatient health-care facilities, 26 27 in Brazil, misoprotol is only approved for hospital use, which requires the hospitalization of women for medical abortion, a more invasive and expensive treatment option. Brazilian medical residents consider some important characteristics for outpatient use as advantages: “does not requires anesthesia”, “it is less invasive”, “can be accompanied”, is “less expensive”. They also do not consider the need of more visits to be a problem. These opinions favor implementing such outpatient treatment choice.

The results of the present study may provide valuable information to those responsible for the orientation of women requesting legal termination of pregnancy and who should decide if they prefer a surgical or a medical procedure, as our results inform which are the most important advantages and disadvantages of medical abortion in the judgment of residents, a very valuable information for the women who will experience this procedure.

## Conclusion

Residents perceived both clinical and personal issues as advantages of medical abortion.

## References

[1] Boland R Katzive L Developments in laws on induced abortion: 1998-2007 Int Fam Plan Perspect 2008 34 03 110 120 10.1363/ifpp.34.110.08 18957353

[2] Diniz D Medeiros M Madeiro A National abortion survey 2016 Cien Saude Colet 2017 22 02 653 660 10.1590/1413-81232017222.23812016 37255138

[3] Sedgh G Bearak J Singh S Bankole A Popinchalk A Ganatra B Abortion incidence between 1990 and 2014: global, regional, and subregional levels and trends Lancet 2016 388(10041):258 267 10.1016/S0140-6736(16)30380-4 27179755 PMC5498988

[4] Diniz D Brito L Ambrogi I Tavares A B Ali M Understanding the sexual and reproductive health needs in Brazil's Zika-affected region: placing women at the center of the discussion Int J Gynaecol Obstet 2019 147 02 268 270 10.1002/ijgo.12924 31314905

[5] Malta M Wells S LeGrand S Seixas M Baptista A Silva C MFP Abortion in Brazil: the case for women's rights, lives, and choices Lancet Public Health 2019 4 11 e552 10.1016/S2468-2667(19)30204-X 31677774

[6] Grimes D A Benson J Singh S Romero M Ganatra B Okonofua F E Shah I H Unsafe abortion: the preventable pandemic Lancet 2006 368(9550):1908 1919 10.1016/S0140-6736(06)69481-6 17126724

[7] Faúndes A Misoprostol: life-saving Eur J Contracept Reprod Health Care 2011 16 02 57 60 10.3109/13625187.2011.561940 21417559

[8] Silva D FO Bedone A J Faúndes A Fernandes A MS Lima e Moura V GA Aborto provocado: redução da frequência e gravidade das complicações. Consequência do uso de misoprostol? Rev Bras Saúde Mater Infant 2010 10 04 441 447 10.1590/S1519-38292010000400004

[9] Faúndes A Bedone A Silva J LP I Fórum interprofissional para implementação do atendimento ao aborto previsto por lei: relatório final Femina 1997 25 01 69 78

[10] Ministério da Saúde Secretaria de Atenção à Saúde Departamento de Ações Programáticas Estratégicas Norma técnica: prevenção e tratamento dos agravos resultantes da violência sexual contra mulheres e adolescentes Brasília (DF) Ministério da Saúde 2010

[11] Faúndes A Araújo M JO Andalaft Neto J Ferreira M FO Relatório final: X Fórum interprofissional sobre violência contra a mulher e implementação do aborto previsto na lei Femina 2007 35 01 55 58

[12] Faúndes A Duarte G A Osis M JD Andalaft Neto J [Knowledge and opinion variations of Brazilian obstetricians and gynecologists face to legal abortion, between 2003 and 2005] Rev Bras Ginecol Obstet 2007 29 04 192 199 10.1590/S0100-72032007000400005

[13] Fiol V Rieppi L Aguirre R Nozar M Gorgoroso M Coppola F Briozzo L The role of medical abortion in the implementation of the law on voluntary termination of pregnancy in Uruguay Int J Gynaecol Obstet 2016 134 01 S12 S15 10.1016/j.ijgo.2016.06.006 28748589

[14] Ganatra B Tunçalp Ö Johnston H B Johnson B R Jr Gülmezoglu A M Temmerman M From concept to measurement: operationalizing WHO's definition of unsafe abortion Bull World Health Organ 2014 92 03 155 10.2471/BLT.14.136333 24700971 PMC3949603

[15] Palma Manríquez I Moreno Standen C Álvarez Carimoney A Richards A Experience of clandestine use of medical abortion among university students in Chile: a qualitative study Contraception 2018 97 02 100 107 10.1016/j.contraception.2017.09.008 28947389

[16] World Health Organization Preventing unsafe abortion Geneva WHO 2018

[17] Kulier R Kapp N Gülmezoglu A M Hofmeyr G J Cheng L Campana A Medical methods for first trimester abortion Cochrane Database Syst Rev 2011 11 CD002855 10.1002/14651858.CD002855.pub4 22071804 PMC7144729

[18] World Health Organization Safe abortion: technical and policy guidance for health systems 2nd ed. Geneva WHO 2012 23700650

[19] Ireland L D Gatter M Chen A Y Medical compared with surgical abortion for effective pregnancy termination in the first trimester Obstet Gynecol 2015 126 01 22 28 10.1097/AOG.0000000000000910 26241252

[20] Laursen L Stumbras K Lewnard I Haider S Contraceptive provision after medication and surgical abortion Womens Health Issues 2017 27 05 546 550 10.1016/j.whi.2017.03.012 28487068

[21] Ministério da Saúde Secretaria de Atenção à Saúde Departamento de Ações Programáticas Estratégicas Prevenção e tratamento dos agravos resultantes da violência sexual contra mulheres e adolescentes: norma técnica 3a ed. Brasília (DF) Ministério da Saúde 2012

[22] Grupo de Estudos sobre Aborto no Brasil Pacagnella R C Bento S F Fernandes K G Araújo D M Fahl I D Fanton T F Knowledge on medical abortion among Brazilian medical residents in Gynecology and Obstetrics Cad Saude Publica 2020 36 36 01 e00187918 10.1590/0102-311x00187918 32049117

[23] R Core Team R: a language and environment for statistical computing [Internet] Vienna R Foundation for Statistical Computing 2017[cited 2017 Mar 6]. Available from:http://www. www.R-project.org/

[24] Ho P C Women's perceptions on medical abortion Contraception 2006 74 01 11 15 10.1016/j.contraception.2006.02.012 16781253

[25] Saurel-Cubizolles M J Opatowski M David P Bardy F Dunbavand A Pain during medical abortion: a multicenter study in France Eur J Obstet Gynecol Reprod Biol 2015 194 212 217 10.1016/j.ejogrb.2015.09.025 26448133

[26] Platais I Tsereteli T Grebennikova G Lotarevich T Winikoff B Prospective study of home use of mifepristone and misoprostol for medical abortion up to 10weeks of pregnancy in Kazakhstan Int J Gynaecol Obstet 2016 134 03 268 271 10.1016/j.ijgo.2016.02.018 27352735

[27] Winikoff B Dzuba I G Chong E Goldberg A B Lichtenberg E S Ball C Extending outpatient medical abortion services through 70 days of gestational age Obstet Gynecol 2012 120 05 1070 1076 10.1097/aog.0b013e31826c315f 23090524

